# Decoding the grasping intention from electromyography during reaching motions

**DOI:** 10.1186/s12984-018-0396-5

**Published:** 2018-06-26

**Authors:** Iason Batzianoulis, Nili E. Krausz, Ann M. Simon, Levi Hargrove, Aude Billard

**Affiliations:** 1 Learning Algorithms and Systems Laboratory (LASA), School of Engineering, École Polytechnique Fédérale de Lausanne (EPFL), Route Cantonale, Lausanne, CH-1015 Switzerland; 2 Center for Bionic Medicine, Shirley Ryan AbilityLab, E Erie St., Chicago, 60611 IL USA; 3 Dept. of Physical Medicine and Rehabilitation, Northwestern University, N Lake Shore, Chicago, 60611 IL USA; 4 Dept. of Biomedical Engineering, Northwestern University, Evanston, 60208 IL USA

**Keywords:** Myoelectric control, Upper limb prosthesis, Pattern recognition, Reach-to-grasp motion

## Abstract

**Background:**

Active upper-limb prostheses are used to restore important hand functionalities, such as grasping. In conventional approaches, a pattern recognition system is trained over a number of static grasping gestures. However, training a classifier in a static position results in lower classification accuracy when performing dynamic motions, such as reach-to-grasp. We propose an electromyography-based learning approach that decodes the grasping intention during the reaching motion, leading to a faster and more natural response of the prosthesis.

**Methods and Results:**

Eight able-bodied subjects and four individuals with transradial amputation gave informed consent and participated in our study. All the subjects performed reach-to-grasp motions for five grasp types, while the elecromyographic (EMG) activity and the extension of the arm were recorded. We separated the reach-to-grasp motion into three phases, with respect to the extension of the arm. A multivariate analysis of variance (MANOVA) on the muscular activity revealed significant differences among the motion phases. Additionally, we examined the classification performance on these phases. We compared the performance of three different pattern recognition methods; Linear Discriminant Analysis (LDA), Support Vector Machines (SVM) with linear and non-linear kernels, and an Echo State Network (ESN) approach. Our off-line analysis shows that it is possible to have high classification performance above 80% before the end of the motion when with three-grasp types. An on-line evaluation with an upper-limb prosthesis shows that the inclusion of the reaching motion in the training of the classifier importantly improves classification accuracy and enables the detection of grasp intention early in the reaching motion.

**Conclusions:**

This method offers a more natural and intuitive control of prosthetic devices, as it will enable controlling grasp closure in synergy with the reaching motion. This work contributes to the decrease of delays between the user’s intention and the device response and improves the coordination of the device with the motion of the arm.

**Electronic supplementary material:**

The online version of this article (10.1186/s12984-018-0396-5) contains supplementary material, which is available to authorized users.

## Background

The loss of a hand impacts all aspects of daily life, including work, recreation and communication [1]. Prosthetic devices can help restore motor abilities lost after amputation, and can improve the quality of life for amputees. Despite these potential benefits, many people with upper-limb amputations do not use a prosthesis [2]. Studies examining the causes of rejection [3–5] report that secondary prosthesis rejection, i.e. after a period of use, is primarily due to dissatisfaction with prosthetic comfort, function or control. Improving prosthesis control systems could thus presumably provide additional functionalities to users and could minimize prosthesis rejection.

Surface electromyography (EMG) has been widely studied as an intuitive human-machine interface for controlling intelligent external devices, such as prosthetic hands [6, 7]. As amputees generally have a limited number of independent EMG sites available for controlling a multi-degree of freedom (DOF) prosthesis, we cannot rely on a one-to-one EMG-to-DOF control. Surgical methods, such as targeted muscle reinnervation (TMR) [8, 9] and regenerative peripheral-nerve interfaces (RPNIs) [10], can enable the control of a larger number of DOFs.

Advanced signal-processing approaches could also be used to control multi-DOF prostheses with fewer independent EMG sites. EMG-based pattern-recognition systems are proposed for estimation of, both independent and simultaneous [11, 12], hand and wrist movements [13–15]. Using extrinsic hand muscles, pattern recognition has effectively classified functional hand-grasp patterns [13] and even individual finger movements [16, 17].

In these previous studies, generally subjects performed muscle contractions while maintaining their arm in a fixed position. However, training a classifier in a static position, as mentioned above, results in lower classification accuracy when the limb is in different positions or performs dynamic motions [18].

**Fig. 1:**
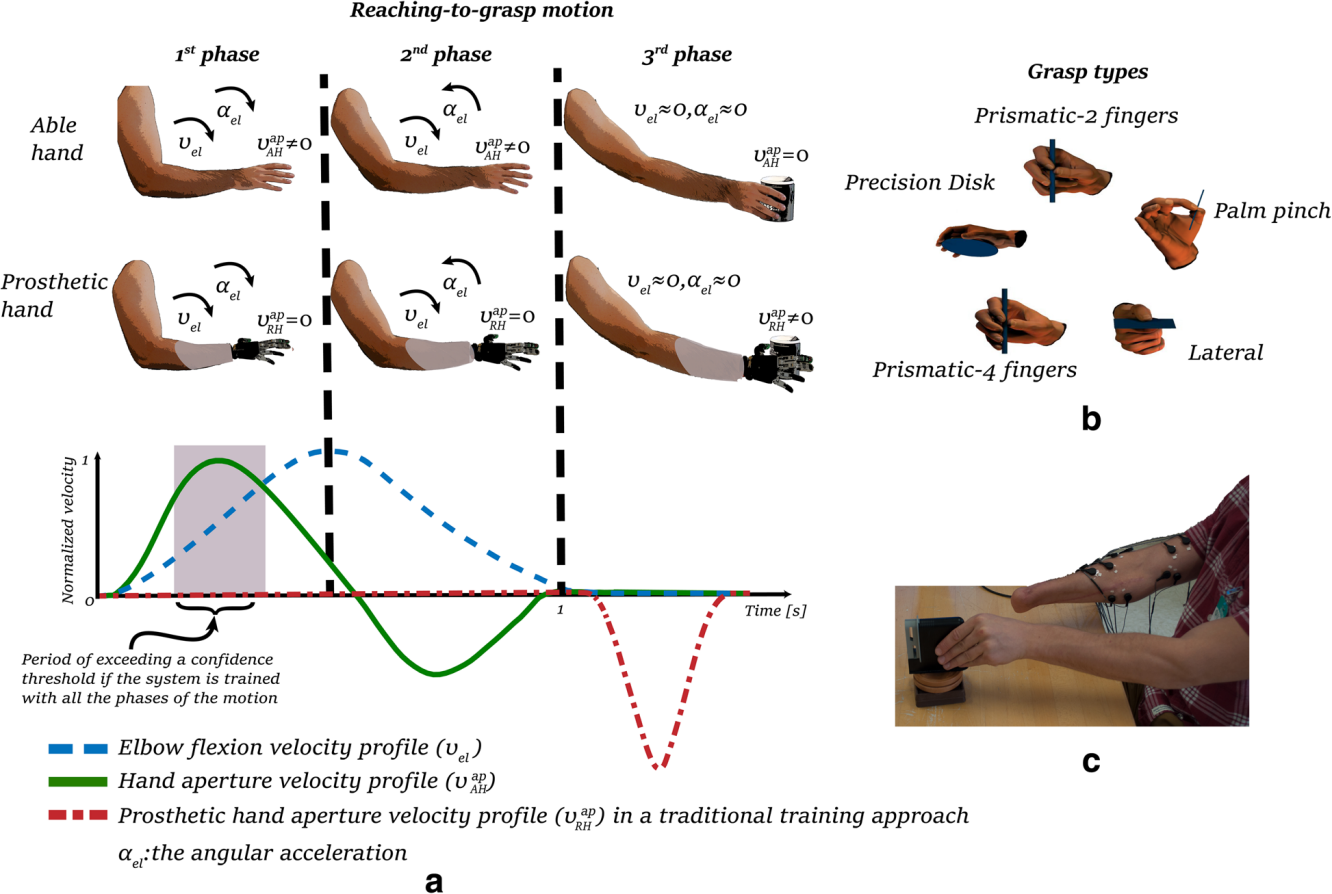
**a** Typical profiles of velocities of the elbow and hand aperture in abled-bodied subject [21, 45, 46] compared to that generated with a traditional prosthetic device, as presented in [25, 26]. During reaching, the aperture of the human hand (solid green line) changes in coordination with the extension of the arm (dashed blue line). In contrast, the prosthetic hand (dash-dotted red line) begins its motion later in the reach-to-grasp cycle, once the elbow is fully extended. In our approach, we separate the reach-to-grasp motion into three phases (denoted by dashed vertical lines) according to the angular acceleration of the elbow joint *a*_*el*_. We distinguish between acceleration, deceleration and rest phases. We present that a pattern recognition system, trained including the reaching motion, could gain efficient prediction confidence early in the reaching motion and, thus, activate faster a prosthetic device. **b** The selected five grasp types used in our classification, following the names and using figures from the taxonomy of [47]. **c** Experimental set-up for training the system with amputee subjects in data recordings. EMG-information from the amputated arm are recorded while the subject performs the reach and grasp motion with his/her intact arm

Reach-to-grasp movements are important activities of daily living that require dynamic motions. A few studies have attempted to decode grasping intention from EMG during reach-to-grasp motions, but only with able-bodied subjects [19, 20]. When reaching to grasp an object, the opening and closing of the hand is in coordination with the motion of the arm [21, 22], see Fig. 1a. More specifically, the human hand opens rapidly in the early stages of the reaching cycle, while the fingers converge gradually to their final configuration [21, 23].

A self-paced reaching motion of an able-bodied hand could take approximately 1 s to complete [19, 24]. In contrast, the activation of prosthetic hands could occur more than one second after the onset of the motion [25, 26] (see Fig. 1a). This makes unnatural the actuation of a prosthetic hand, lacking the natural arm-hand coordination. It also slows down the reach-to-grasp motion. It is crucial for prosthetic devices to react promptly to human intentions to enable for natural and intuitive operations [27]. To convey a seamless coordination between the device and the residual arm, it is important for the device to identify the grasping intention during the reaching phase.

In our previous work [19], we show that detection of the grasp type in synchrony with the reaching motion could enable smooth coordination of hand closure with the reaching motion, thus providing a more natural and seamless motion of the arm and a robotic hand. In our approach, the classification performance is related to the occurrence of hand pre-shape during reaching motions, following the natural pre-shape phase as documented in [23, 28]. Our prior study was limited to able-bodied subjects. Here, we extend this approach to decoding residual EMG in individuals with a below-elbow amputation. We compare the performance of four different classifiers: LDA, two SVMs, and an Echo State Network (ESN). Additionally, we explore a relationship between classification performance and the phases of the reach-to-grasp motion.

## Methods

### Experimental protocol

Eight able-bodied subjects (6 males and 2 females 25–32 years old) with no known neurological or physical deficits and four unilateral transradial amputees participated in the experiment. All able-bodied subjects were right-handed and performed the experiment with their dominant hand. All subjects were naive to pattern recognition control with the exception of three of the amputee subjects. Two of the amputee subjects had undergone a TMR surgery. Table 1 presents demographic information about the amputee subjects.

**Table 1:** Demographic information of the four transradial amputatee subjects

Subject	TMR	User of myoprosthesis	Age	Years since amputation
TR1	Yes	No	25	7
TR2	No	myoelectric prosthesis	53	38
TR3	Yes	myoelectric prosthesis	51	2
TR4	No	myoelectric prosthesis	68	> 30

TR1 and TR3 underwent a TMR operation for neuroma pain, not for improving prosthetic control. TR1 is not a user of a myoprosthesis due to financial reasons

During the experiment, both the able-bodied subjects and the amputee subjects sat in front of a table, facing a computer screen, with their elbows at a 90°angle. The able-bodied subjects had their right hand closed on the table and they were asked to reach the object and grasp it with a predefined grasp type and to mentain the same wrist orientation for all grasp types. Custom computer software, called Control Algorithms for Prosthetics Systems (CAPS) [9], prompted users to initiate the reach-to-grasp motion and to go back to a resting position. Subjects performed the motion at their own pace. They were tasked to reach and grasp an object placed 30 cm away from the initial position of their hand. Once they had reached the objects, they were asked to remain in the same posture until they receiving a cue from CAPS to go back to rest position. The duration of each trial was 4 s with a 10 s rest between trials, to avoid fatigue. All subjects performed 30 trials for five grasp types, resulting in 150 trials in total.

In the experiments with the amputee subjects, the subjects were asked to reach the object and grasp it with their intact hand while trying to replicate the motion with their phantom limb, see Fig. 1c. These subjects started their self-paced motion when cued by the experimenter. Whenever a subject perceived an irregular or unexpected muscle contraction, the experiment was paused and the trial was repeated. Regular breaks were taken in order for the subjects to relax from the stress and effort of contracting their phantom limb. All the amputee subjects were able to complete the experiments.

### Apparatus and pre-processing

Custom computer software [9] was used for signal acquisition, with EMG signals acquired at 1000 Hz with a 30–350 Hz band-pass filter using TI ADS1298 biosignal amplifiers. The EMG activity of 12 muscles was recorded: *Trapezius, Deltoid Anterior, Deltoid Medial, Deltoid Posterior, Biceps Brachii long head, Triceps Brachii long head, Brachialis, Flexor Digitorum Superficialis, Extensor Digitorum Communis, Flexor Carpi Ulnaris, Extensor Carpi Ulnaris, Flexor Carpi Radialis* (seven muscles of the upper arm and five muscles of the forearm). To construct a linear envelope, full-wave rectification was performed, followed by smoothing with a low-pass seventh-order Butterworth filter with cut-off frequency at 20Hz. At the end of this step, each channel was normalized by the maximum value recorded in the trials. A goniometer was placed on the subjects’ elbow for measuring the onset and extension of the elbow. Please see the Additional file 1 for the exact location of the EMG sensors.

### Phases of the motion

As illustrated in Fig. 1a, during natural reach-to-grasp motion, the opening and closing of the hand is coordinated with the extension of the elbow [21, 22]. Typically for able-bodied subjects, the hand opens rapidly in the early stages of the reaching motion and decreases its velocity while approaching the object and reaching the final configuration [23]. The hand’s velocity peak occurs before the peak velocity of the elbow extension [21]. Thus, the hand reaches its final grasp-posture after the peak velocity of the elbow extension. Because EMG recordings from upper and lower arm muscles encapsulate information on the hand motion, the EMG patterns will likely differ in the different phases, specifically before and after the elbow extension velocity peak. Taking inspiration from this behavior, we divided the reach-to-grasp motion into three phases with respect to the extension of the elbow joint. The first phase is defined as the interval from motion onset (i.e. when the angular velocity of the elbow joint exceeds a velocity threshold) until the angular velocity of the elbow reaches its maximum. The second phase is the interval between the aforementioned maximum angular velocity and the end of the reaching motion (i.e. when the angular velocity of the elbow drops below a velocity threshold). We define the third phase as the phase after the completion of the elbow extension. More specifically, we selected 25% of the duration of the reaching motion selected after the velocity drops below a threshold. The velocity threshold was set at 10% of the maximum angular velocity recorded for each subject.

We normalized the time of the duration of the reaching cycle. The reaching cycle corresponds to the time interval between the motion onset and the end of the extension of the elbow, i.e. when the angular velocity of the elbow drops below the velocity threshold. We performed a one-way multivariate analysis of variance (MANOVA) on the average values of the 12 EMG channels over the three phases for each grasp type. The Wilks Lambda test and the Pillai-Barlett Trace test were used to compare the results to a significance level of 5% (*a*=0.05). We present the results in “Results” section.

To further investigate the three phases, we grouped the EMG signals of the classes together for each phase. The signals were divided into sliding time windows and the average activity of each channel was extracted, creating a vector of N elements (N corresponds to the number of EMG channels). A principal component analysis (PCA) was performed with the data from the third phase and the two remaining phases were projected into the new hyperplane. The distribution of the data on the first two principal components was fitted to Gaussian Mixture Models (GMMs) for each phase, and the number of Gaussian components was optimized by the Bayesian Information Criterion (BIC). We performed an analysis on the complete muscle set (*N*=12) and an analysis using only the muscles of the forearm (*N*=5).

### Classification methods for decoding the grasping intention

The preprocessed EMG signals were analyzed using a sliding time-window of 150 ms with an increment of 50 ms. The time window and increment lengths were chosen to be between the preferred values for an online implementation, as suggested in [29]. We used no dimensionality-reduction method (such as PCA) in this step. For each grasp type, 10 trials were randomly selected as the testing set. The remaining 20 trials of each grasp type constituted the training and validation sets. A four-fold cross-validation was performed to optimize the hyper-parameters for each classification method.

The classification accuracy of four classification methods was compared, namely for an LDA classifier, an SVM with linear kernel, an SVM with a Radial Basis Function (RBF) kernel and an ESN. For each classification method, one classifier was trained per subject. We did not attempt any inter-subject training. We inserted the classification outcome of each time window into a Majority Vote (MV) algorithm, that uses a buffer of 0.5 s history to predict the winning class.

In the cases of LDA and SVM, we extracted three features for each time window and introduced them into the classifier. Following previously described methods for EMG pattern recognition [30], we chose three features; the average activation of each time window, its waveform length, and the number of slope changes. In the case of ESN, we did not perform any feature extraction, treating the problem as a multidimensional time-series problem.

#### Linear discriminant analysis

LDA is one of the most commonly used classification algorithms for biomedical signals due to its performance and robustness. LDA finds a linearly optimal combination of the features in order to separate between classes. A fitting function estimates the parameters of a Gaussian distribution for each class and finds the probability of each point belonging to a class. Despite the linear nature of LDA, it has been shown to perform well in the classification of EMG signals [31].

#### Support vector machine

We tested two types of kernels, i.e. a linear kernel and an RBF kernel. In the case of the linear kernel, after a grid search, we optimized the penalty factor *C*. Likewise in the case of the RBF kernel, we optimized by a grid search both the penalty factor *C* and the *γ* parameter.

#### Echo state networks

ESN [32] is an effective recurrent neural network (RNN) that has attracted substantial interest due to its performance in time-series [33, 34]. The core of ESN is a large fixed reservoir. The reservoir contains a large number of randomly and sparsely connected neurons. The determination of the readout weights is the only trainable part; the weights can be obtained simply by linear regression. The necessary and sufficient condition to generate the echo state is based on information from the dynamic reservoir, such as the spectral radius of the internal weight-matrix. We optimized the three hyper-parameters; number of neurons, spatial radius and regularization parameter, by a grid search.

### Physical prosthesis control

For the purpose of the online implementation, we used the RIC hand [35], a prototype prosthesis with two degrees of actuation, that is able to perform two grasping postures: hand open, power grasp and prismatic-2 fingers. Due to its design, the RIC hand can offer access to a lower level control of the actuators. In this control scheme, we could control the actuators directly hence avoid any delays that arise from using control interfaces offered by commercial prosthesis. We mounted the prosthesis on a socket fitted to the user’s residual forearm and a goniometer was placed on the elbow joint to record arm extension. We collected 12 EMG signals from the arm, preprocessed and classified them in real time and we inserted the classification output into a majority vote algorithm. The buffer of the majority vote was 0.5 s. Once the majority vote confidence exceeded a threshold of 0.5, i.e. more than half of the votes belonged to the same class, the corresponding command was sent to the prosthesis.

One subject, TR4, participated in a real-time control experiment. During the training phase, we cued the subject to perform 20 reach-to-grasp motions for each trained grasp type. The collected EMG signals were used to train two SVMs with RBF kernels: the first classifier used EMG from all the motion phases, whereas the second classifier was trained with EMG collected after arm extension (only the third phase). During the testing phase, the subject performed two sets of 20 reach-to-grasp trials for each classifier with the prosthesis turned on. Prior to the testing phase, the subject controlled the prosthesis for 10–15 min to familiarize themself with the device control. We used two metrics to compare the performance of the classifiers: the classification accuracy, and the time to reach a 0.5 majority-vote confidence level. We performed a two-sample t-test to validate the null hypothesis and to determine if there were significant differences between the results from the two classifiers.

## Results

### Phases of the motion

**Fig. 2:**
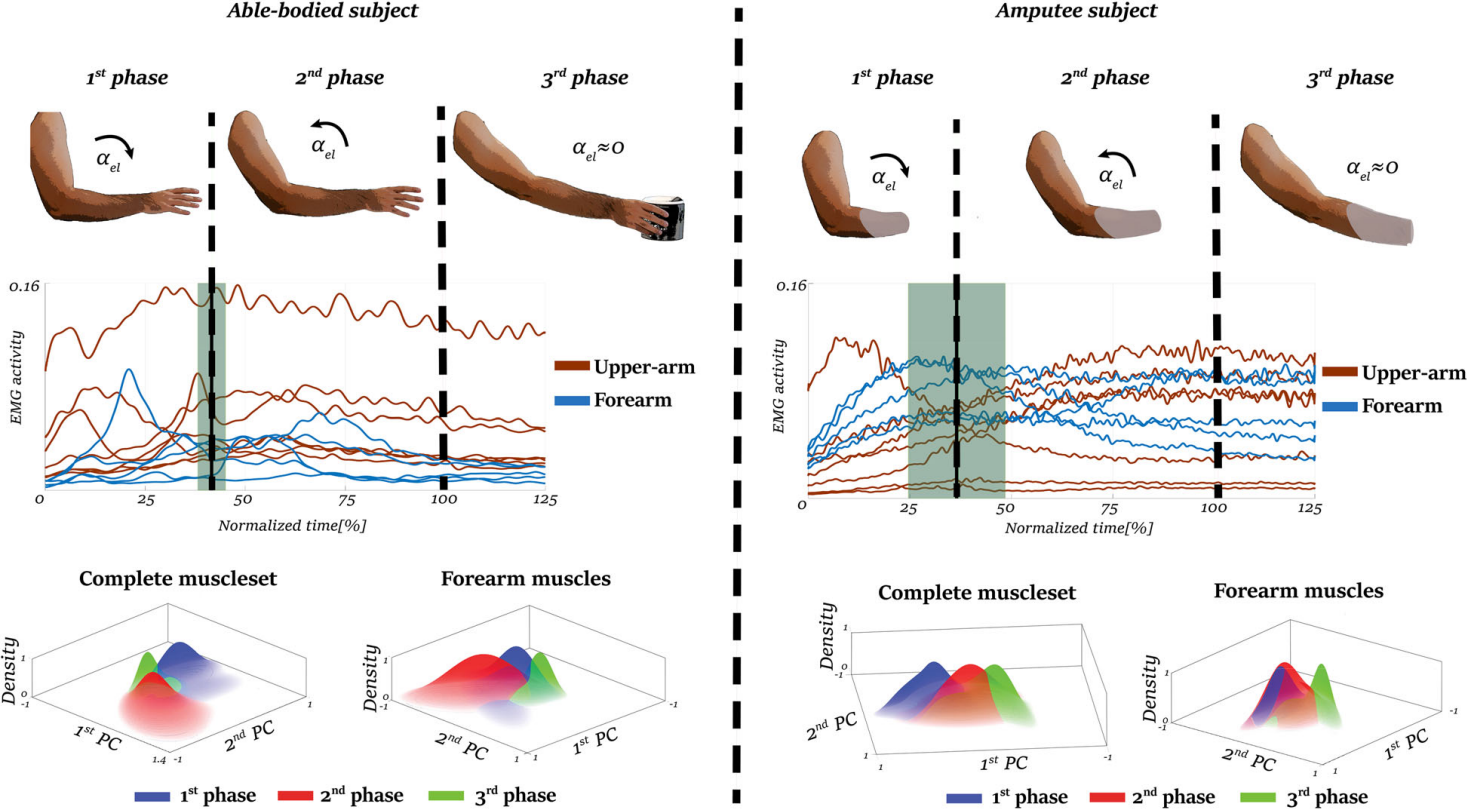
Representative examples of the EMG activity and the phases of the motion of the able-body subject 4 (left) and the amputee subject TR4 (right). The graphs in the middle correspond to the linear envelope of the EMG signals of the upper-arm (red lines) and the forearm (blue lines). The grey shadow areas correspond to the standard deviations of the timings where the shifts between the phases occurred. The graphs on the bottom of the figure show a representation with Gaussian Mixture Models (GMMs) of the EMG activity of the three phases projected on the first two components of third phase after performed Principal Component Analysis (PCA). The analysis was performed on the complete muscle set (*N*=12) and when using only the muscles of the forearm (*N*=5). The GMR representation shows limited overlap between the three phases, indicating differences on the EMG activity of the phases. Occasionally, an extended overlap occurred between the first and second phases as presented in the bottom-right graph. However, the third phase had rarely overlapped with any of the other two phases. Please see the Additional file 1 for more information regarding the EMG activity and its GMM representation for all the participants

To examine the muscle-activation patterns during the reaching motion, we divided the recorded EMG signals in two groups: muscles of the forearm and muscles of the upper arm. Figure 2 presents representative examples of the average EMG activity of each muscle group in normalized time; the blue color corresponds to the muscles of the forearm and the red color corresponds to the muscles of the upper arm. The vertical dashed lines highlight the average time of the shift from the 1^*st*^ phase to the 2^*nd*^ phase, with the green shaded areas corresponding to the standard deviation of that shift. We calculated these events from the kinematic data recorded by the goniometer. The mean reaching time varied between 0.97±0.16*s* to 1.26±0.3*s* for able-bodied subjects and from 1.13±0.23*s* to 1.7±0.3*s* for amputee subjects

We identified the maximum elbow-joint angular velocity at 30–45% of the reaching motion for all the participants. We found no significant difference between able-bodied and amputee subjects regarding the timing of maximum elbow-joint angular velocity (*p*=0.55, *t*−*value*=1.45). The activation pattern of the distal muscles (muscles of the forearm) differed between amputee and able-bodied participants. In particular, the activation of the distal muscles in able-bodied subjects occurred earlier than in amputees. The muscular activity of the forearm muscles of able-bodied subjects reached a peak from 20–60% of the motion, decreasing as the motion is came closer to completion.

The EMG activity of forearm muscles of the amputee subjects increases gradually during the reaching. Whereas, the proximal muscles remain at a constant level of activation after the maximum angular velocity is reached. This difference in activation timing could have an effect on classification performance.

We compared the average activity across the three phases with a one-way MANOVA. As it rejected the null hypothesis, we found significant differences between the phases (*p*<0.01, *Degrees of Freedom (DoF)* =2) for all the subjects (able-bodied and amputees). Figure 2 presents the Gaussian models of the phases on the first two principal components for the complete muscle-set and the muscles of the forearm, respectively.

Although some models partially overlap, they have different mean values for all subjects, regardless of the muscle-set. In able-bodied subjects, the third-phase models are concentrated around the origin and have standard deviations smaller than the other phase models.

For amputees, the third-phase models are concentrated around the origin, similar to the able-bodied results. however, these models cover an area larger than the corresponding models for the able-bodied subjects. Also, a larger overlap was found between the models first and second phases for all amputee subjects, with larger distance from the models of third-phase.

We compared the average muscle activity during the three phases for each class (i.e. grasp type) by performing a one-way MANOVA. The one-way MANOVA failed to reject the null hypothesis (*p*<0.001,*DoF*=2), indicating significant differences between the means of the phase models for all the subjects. The significant differences between the three phases show that the data from all classes in the space change continuously, thus reducing the ability for a classifier to generalize across the three phases if it is trained on only one of them.

### Decoding the grasping intention

**Fig. 3:**
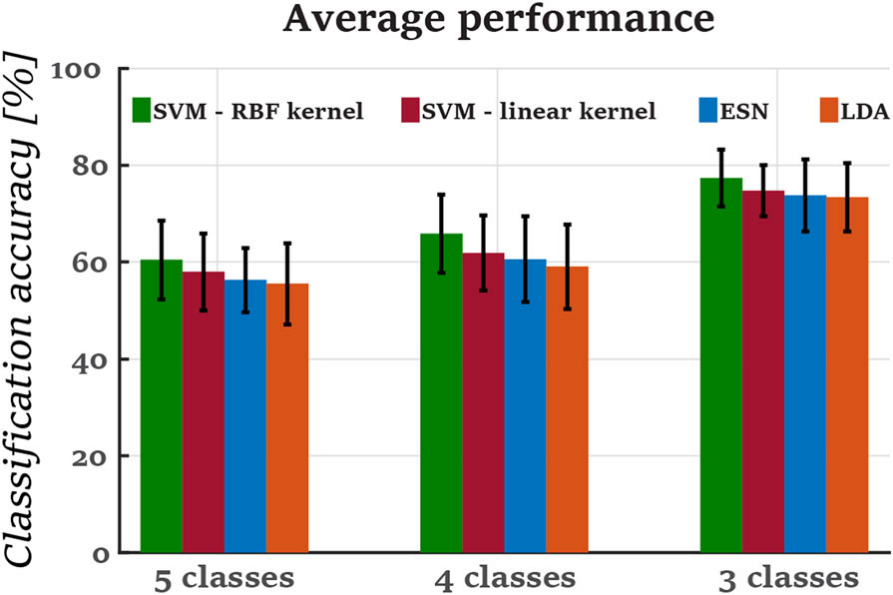
The average performance of all the classification models through out the whole trajectory of 2*s* among all the subjects.No significant differences were noticed between the performances of the classifiers for each group of classes(*p*=0.7, *p*=0.5 and *p*=0.8 for 5, 4 and 3 classes respectively

As stated previously, we compared the performance of four classifiers (LDA, SVM with linear kernel, SVM with RBF kernel and ESN). Figure 3 presents the average classification accuracy of each classifier over a time interval of 2*s*. After performing an analysis of variance (ANOVA) for a significance level of 5%- *a*=0.05, we did not notice any significant differences between the classifiers’ performance for each group of classes ({*p*=0.7,*F*−*value*=0.43}, {*p*=0.5,*F*−*value*=0.97} and {*p*=0.8,*F*−*value*=0.35} for 5, 4 and 3 classes respectively). However, the SVM classifier with the RBF kernel performed better than the other classifiers with 60.45±8.2*%*, 65.82±8*%* and 77.4±5.88*%* classification accuracy for 5,4 and 3 classes, respectively, but this difference was not significant. This was followed by the SVM with the linear kernel, the ESN and the LDA. As the SVM-RBF classifier achieved slightly better performance, the rest of the results correspond to the performance of this classifier.

**Fig. 4:**
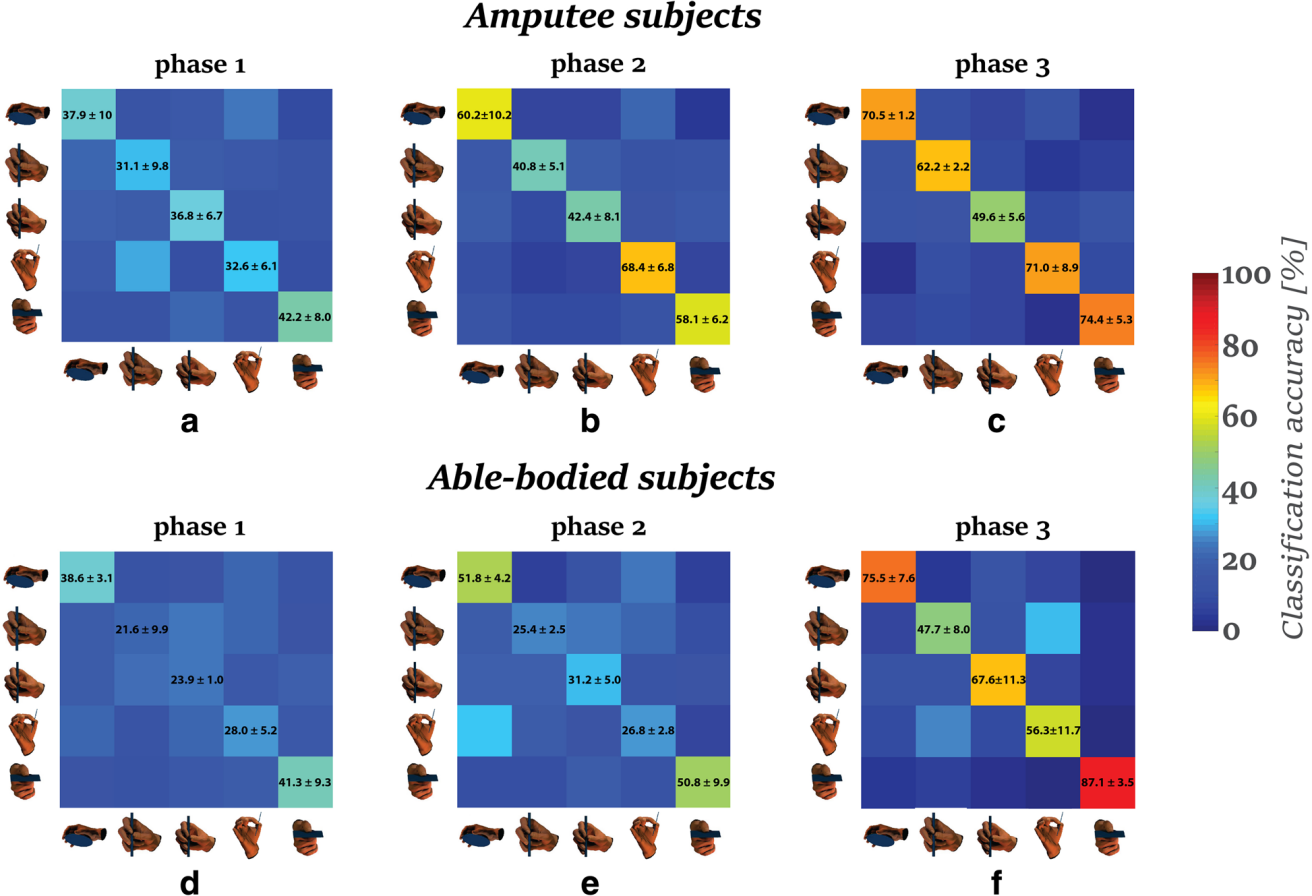
The confusion matrices for all the motion phases (phases 1, 2 and 3). The confusion matrices present the average classification accuracies and their standard deviations for the five grasp types. The matrices on the top correspond to the classification performances among the amputee subjects while the matrices on the bottom correspond to the classification performance of the able-bodied subjects. The horizontal axis of the confusion matrices correspond to the predictions while the vertical axis correspond to the ground-truth. The color of the tile was assigned according to the colormap of the classification accuracy on the right. The grasp types are represented by the corresponding figures as it is shown in Fig. 1

Figure 4 presents the classification performances of the five grasp types in each of the three motion phases. Poor classification performance occurred during the first phase in both amputee and able-bodied subjects. Accuracy improved in the subsequent two phases (see Fig. 4a and d). Grasp types; precision disk, palm pinch and lateral grasp, yielded the best performance in second and third phases for amputee subjects (see Fig. 4b and c). The lateral grasp improved from 58.1±6.2*%* in the second phase to 60.45±8.2*%* in the third phase. Accordingly, the precision disk and palm pinch increased from 60.2±10.2*%* to 70.5±1.2*%* and 68.4±6.8*%* to 71±8.9*%*, respectively. The precision disk and lateral grasp types had the best classification accuracy for the able-bodied subjects as well (see Fig. 4e and f). These grasp types’ performances increased from 60.2±10.2*%* and 50.8±9.9*%* in the second phase to 75.5±7.6*%* and 87.1±3.5*%* in the third phase, respectively. We noticed the worst performance in the prismatic-4 fingers for the amputee subjects, with 49.6±5.6*%* in the third phase (Fig. 4c), and in prismatic-2 fingers for the able-bodied subjects, with 47.7±8*%* in the third phase (Fig. 4f). The prismatic-2 fingers and palm pinch were misclassified for one another in the third motion phase for the able-bodied subjects about 25−30*%* (see Fig. 4f). This indicates that the muscular activity during the preshaping of the fingers is similar for these grasps types. The reason for this could the similarity of the two grasp types as they differ mainly on the configuration of the middle finger.

**Fig. 5:**
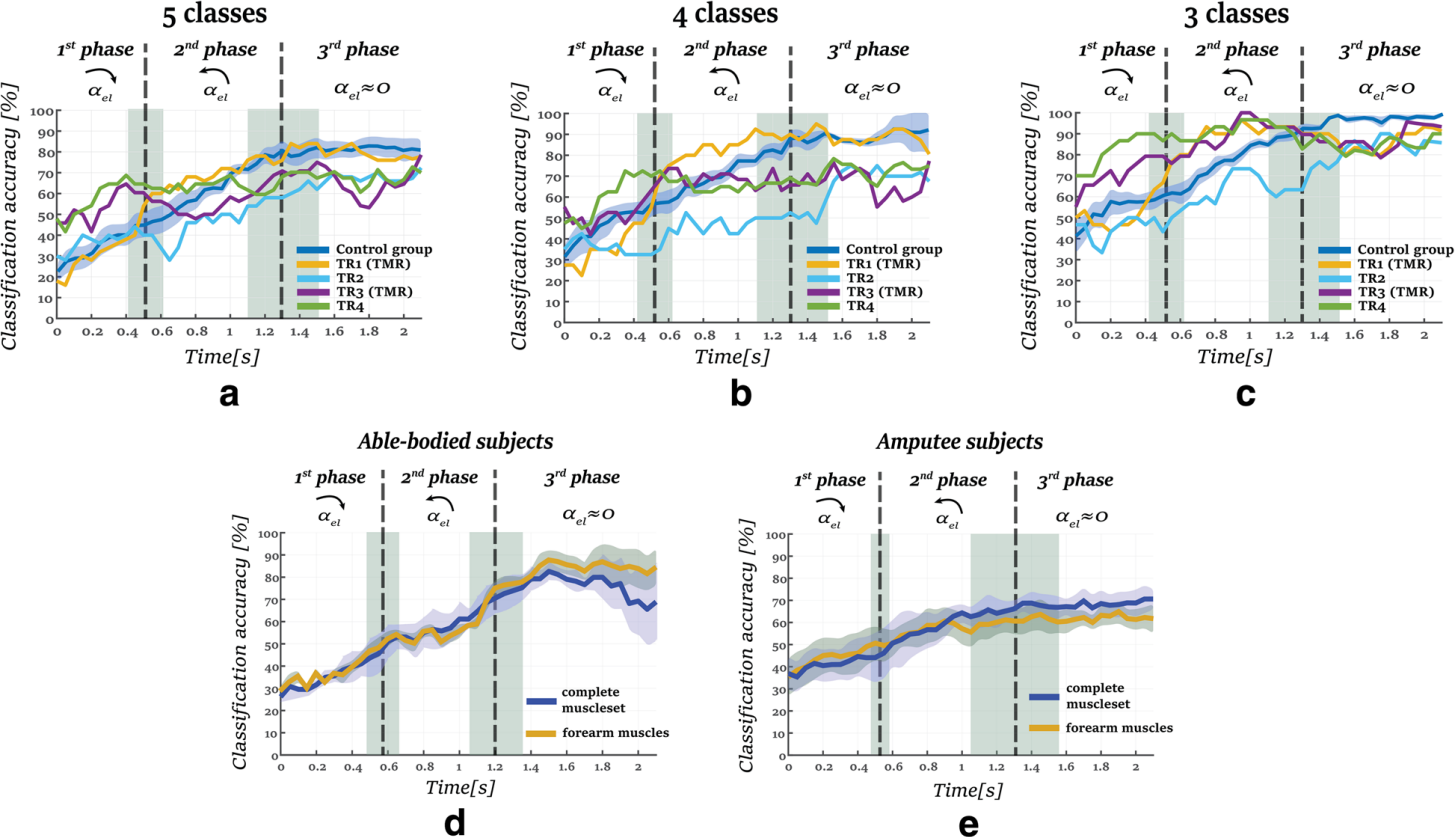
The evolution of classification performance and standard error through time for 2*s* after the motion onset. The vertical dashed lines correspond to the average moments of the shifts of between the phases while the shaded areas present the corresponding standard deviations accordingly. Figures **a**, **b** and **c** present the evolution of the classification performance of the control group and the amputee subjects for 5, 4 and 3 classes accordingly. Figures **d** and **e** compare the classification performance on 5 classes when using the forearm muscles and complete muscle-set as an input to the classifier a_e_l correspond to the angular acceleration of the elbow joint. The arrows above the angular acceleration represent its direction

Figure 5a-c presents the evolution of average classification performance of the control group, that consist of the eight able-bodied subjects, and the individual performance of all the amputee subjects, until 2 s after the motion onset. For the cases of 4−grasp and 5−grasp types, the classification performance of each classifier follows the same profile: poor classification performance in the first phase of the motion, and the performance increases as the hand approaches the object. Subject TR1 achieved the best performance of all amputee subjects, with performance comparable to that of the able bodied subjects, whereas TR2 had the lowest performance. TR3 and TR4 exceeded the level of 60–70% in the accuracy at the end of the first phase and the beginning of the second phase, and they stayed at this level until the end of the third phase.

In the case of 3−grasp types, TR3 and TR4 appeared better performance than the control group in the first phase, reaching 80% of accuracy during the shift to the second phase. In the second phase, the performance among the subjects TR1, TR3 and TR4 reached an accuracy of 90±10*%*, which is higher than the corresponding performance of the control group (76±20*%*). The classification accuracy in the third phase for the these amputee subjects stayed above 80%, though lower than the control group (95±5*%*).

*Reducing the number of channels*

In this section, we compare classification performance for when using only the forearm EMG with that of using the complete muscle set. An SVM classifier with an RBF kernel was trained for each muscle set: the complete muscle set and the five muscles of the forearm. Figure 5d-e present the evolution of the classification accuracy for a duration of 2 s. As shown, using fewer EMG sites led to decreased performance for the amputee subjects. From the end of the second phase and afterwards, the average classification accuracy decreased significantly from 67.2±8.4*%* to 60±8.2*%* when predicting among 5−grasp types (*p*<0.01, *t-value*=4.33). Although the reduction of the EMG sites available had a large impact on the performance of amputee subjects, when using only the muscles of the forearm the performance of able-bodied subjects was higher than when using the full muscle set.

*Comparing the performance of TMR versus non-TMR subjects*

In this section, we compare the classification performance between TMR subjects (TR1 and TR3) and non-TMR subjects (TR2 and TR4). In the case of 5 classes, the average classification accuracies and standard errors in the first, second and third phases of the TMR subjects are 44.1±10.1*%*, 62.7±86.5*%* and 74.9±6.1*%*, respectively. The corresponding performances of the non-TMR subjects in the three motion phases are 47.6±8.9*%*, 54.9±7.5*%* and 67±2.5*%*. The classification accuracies are at the same level also in the case of 4 classes. More specifically, the average classification accuracies and standard errors of the TMR subjects are 45.5±8.9*%*, 76.45±6.4*%* and 77.7±8.4*%* in the first, second and third phase, respectively. The corresponding performances of the non-TMR subjects in the three motion phases are 48.33±10.7*%*, 58.3±6.9*%* and 77.72±8.6*%*. In the case of 3−grasp types, both the TMR and non-TMR groups have improved accuracies with respect to the other two cases (5 and 4 classes). In the first and third phase, the average classification accuracies of the non-TMR group are at the same level as the TMR group; 68.6±8.8*%* and 64±14.4*%* for the first phase and 87.6±3.4*%* and 83.6±4*%* for the third phase, respectively. However, the accuracy of the TMR subjects exceeds the one of the non-TMR subjects in the second phase; 90.2±4.6*%* and 77.8±10.9*%*, respectively. As stated above, TR1 (a TMR-subject) has the better performance among the amputee subjects.

### On-line evaluation

To demonstrate the usability of our proposed approach for controlling a prosthetic hand, we present an on-line implementation of our approach. We followed the protocol described in “Physical prosthesis control” section. One amputee subject took part in this validation. The subject performed reach-to-grasp motions while commanding the device to close in one of two grasp types: a power grasp or a prismatic-2 fingers grasp. In total, the subject performed two sets of 20 trials for each training approach: training over all phases or training only over the third phase.

**Fig. 6:**
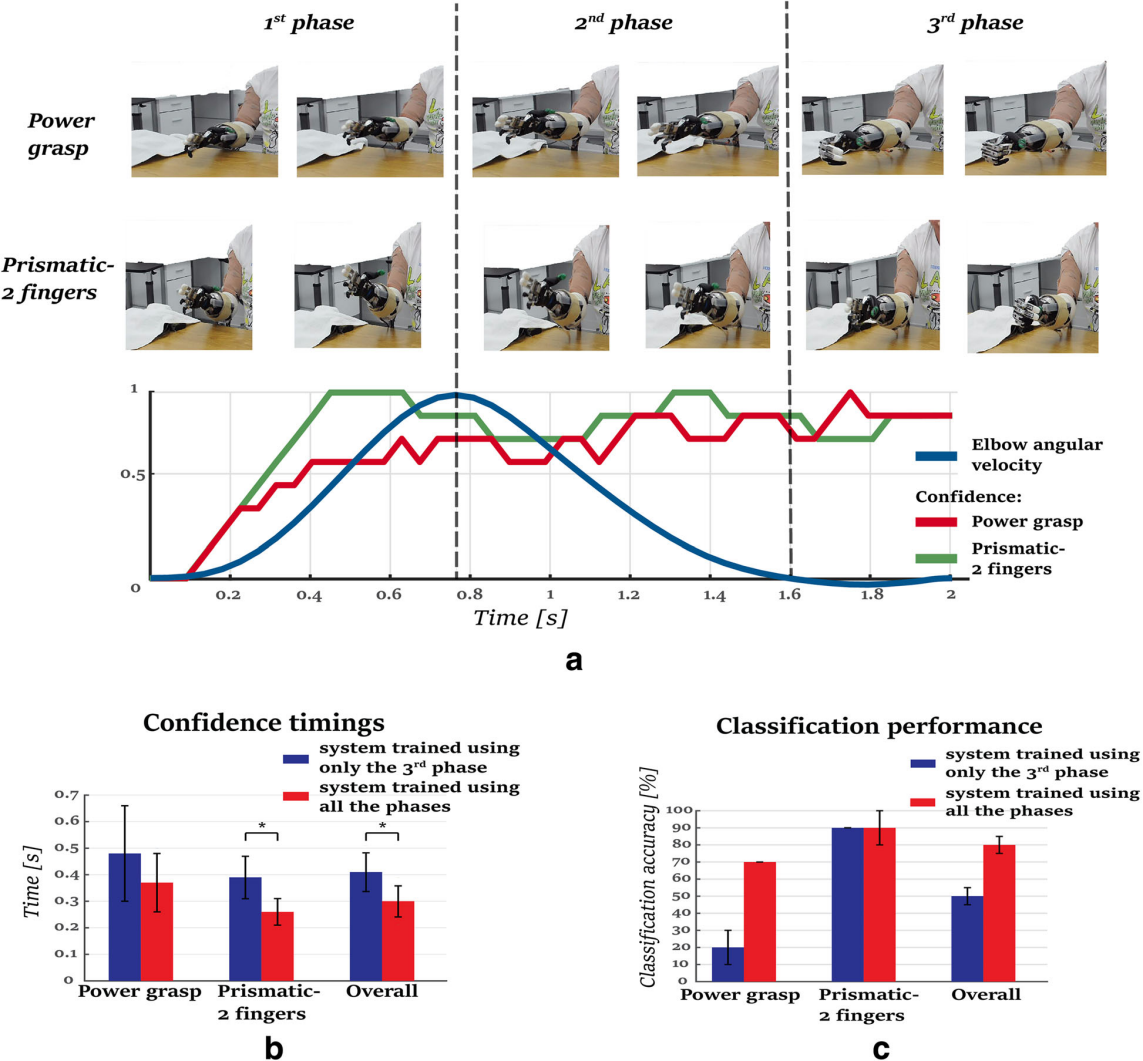
The results of the on-line evaluation. a) Screen-shots of two examples of the on-line implementation showing an activation of the prosthetic device during the second phase. The graph presents the confidence of the majority vote with the elbow’s angular velocity profile. b) the average time and standard deviations until the confidence threshold was reached for the correctly classified trials. The pattern recognition system exceeds the confidence threshold of 0.5 significantly faster (*p*=0.019) when trained including all the phases of the motion. c) the classification accuracy of the testing phase of the on-line evaluation. The pattern recognition system presents better accuracy when trained including all the phases of the motion

An SVM with an RBF kernel was trained off-line, while the testing was performed on-line, where the subject performed 20 reaching motions for each of the aforementioned training approaches. As soon as the classifier reached the confidence threshold of 0.5, the corresponding motor commands were sent to the prosthetic hand to drive the fingers to their desired final posture. We assessed the performance through two metrics: classification accuracy and time to generate a confident prediction on grasp type. Results are shown in Fig. 6b-c.

When trained with all three phases of the motion, the pattern recognition system showed a higher performance in terms of the overall classification accuracy compared to the one that used only the third phase. The overall classification accuracy, when using all three phases for training the system, was 80±5*%* whereas the corresponding accuracy when the system was trained only with the third phase was 55±5*%*, see Fig. 6c. When using only the third the system identified poorly the power grasp type, despite having similar performance for the prismatic-2 fingers grasp type.

The system that trained on all 3 phases was also faster at delivering a robust prediction. On average, it offered a confident prediction 25–40% earlier than the system trained only on the 3rd phase. For the correctly classified trials, when the system trained over all the phases the time needed to exceed the confidence level was significantly lower(*p*=0.0194, *t-value*=2.49) than when it used only the third phase, 0.3±0.10 and 0.42±0.12*s*, respectively (Fig. 6b). The pattern recognition system trained with all the phases reached the confidence threshold at 0.26±0.04*s* for the prismatic-2 fingers grasp, which was significantly faster (*p*=0.003, *t-value*=3.49) than the system trained only with the third phase. Regarding the power grasp, the system trained with all the phases reached the confidence threshold faster, but not significantly (*p*=0.3841, *t*−*value*=0.93), 0.37±0.13*s* than 0.48±0.18*s*, respectively. A video of the on-line evaluation is presented in the Additional file 2.


Additional file 2: The video presents the motivation of this study, the experimental protocol of the off-line analysis as well as an illustration of the models of motion phases and the real time evaluation of the approach. (MP4 54,685 kb)


## Discussion

We present an approach for decoding the grasping intention during reach-to-grasp motions. Although the classification results for our proposed approach are comparable with previous studies [31, 36, 37], it is different for two main reasons. Previous studies examine the classification performance of different hand gestures, including wrist motion, hand open/close, a small number of grasp types (2-4) and in some cases the resting condition [38–40]. Whereas, we focus only on grasping gestures with different finger configurations. Most importantly, previous studies examined static hand-gestures, whereas we investigate the classification of EMG activity during dynamic motions. The inclusion of the reaching motion in the training procedure increases classification performance and confidence, enabling faster activation of the prosthetic device while yielding a seamless and intuitive interaction with the user.

When a hand reaches for an object, the velocity and acceleration profile of the motion are coordinated with the motion of the fingers and the wrist, and the fingers function in a synergistic manner [28, 41]. It is shown that the reach-to-grasp motion consists of many components [42, 43]. Specifically, the motion can be separated into two phases; (1) the reaching phase, when the hand approaches the object while the fingers are pre-shaping [43],and (2) grasping phase, where the hand has traveled the distance to the object and the fingers have taken their final form. This gradual molding of the fingers is revealed through different patterns of muscle activation visible during the reaching motion, which we noticed this in our analysis on able-bodied subjects. Although we cannot observe preshaping in amputees, we assume that this pattern of muscle activation would be preserved partially and would be revealed through different patterns of muscle contractions, as we progress in reach-to-grasp movement.

Taking inspiration from human behavior, we examine the classification performance with respect to the velocity of elbow extension. In particular, we segment the reach-to-grasp motion into three phases: (1) the first phase- where the velocity of the motion increases, (2) the second phase- where the velocity of the motion decreases and (3) the third phase- when the reaching motion is complete. As the average activity of the EMG signals between the three phases is significantly different, training a classifier with only one phase could increase the difficulty of generalizing over the three phases. To highlight these differences, we model the first two principal components with Gaussian Mixture Models (GMMS) for each phase and show phase models occupy different spaces and they only partially overlap (Fig. 2). Hence, classification during different phases of the reaching motion could reduce the variability of the EMG signals, thus increasing the classification accuracy. The lack of motion after the contraction of the muscles could lead to different EMG patterns. As shown in Fig. 2, amputee subjects contract their forearm muscles even in the latter stages of the reaching motion, whereas the EMG of able-bodied subjects converges to lower levels in the final stages. Furthermore, in the case of able-bodied subjects, the fingers pre-shape during the early stages of the reaching motion [43] which results in earlier activation of the forearm muscles. As no pre-shape occurs in transradial amputees, they potentially contract the muscles but solely to close their phantom hand. This could lead to high accuracies in the prediction of the grasp type, even from the first phase with a smaller number of grasp types, as presented in Figs. 5c and 6c.

Therefore, to increase the efficiency of the classification approach, it is important to look into the patterns of the muscular activation. The authors in [39] point out that the muscle activation differs with respect to the arm position and that examining the EMG patterns is important. In our work, we elaborate on the EMG pattern during reach-to-grasp motions, both on able-bodied subjects and individuals with amputation.

In our real-time evaluation, we intend to highlight the negative impact that the lack of good classification over the entire duration of the reaching motion could have in the natural coordination of motion of the prosthesis with the arm. More specifically, we compare the performance of a classifier when it is trained only with one phase (i.e. the third motion phase) against our approach that takes the overall motion into account. Previous approaches [13–15] train a pattern recognition system while maintaining the arm in a fixed position while monitoring the contraction of the muscles. This arm configuration is similar to our third phase, where the extension is complete and the arm remains in the same position. Our results show that the muscle contractions when having the arm fixed are different from the contractions when the arm extends hence the pattern recognition fails to generalize. This leads to lower classification accuracy that results in a slower reaction of the prosthesis. This outcome is aligned with the findings of [38], where a classifier that takes into account different arm positions outperformed a single-position classifier. The benefits of a dynamic training protocol are also shown in [40]. Our work is complementary to these approaches in that it focuses on the timing of classification. As in [40], we address the problem of dynamically estimating the grasp type. To reduce the time needed for reaching a sufficient classification confidence, so as to provide faster reaction time, we focus on combining detection mechanisms.

Relating muscle activation of amputee subjects to the classification accuracy in Fig. 2, we noticed that as the activation level increases, performance also increases. The evolution of the classification accuracy follow the same trend on all the subjects: lower classification in the first phase an higher in the second and third phase (Fig. 5). Although it seems that forearm muscles are most important for classification performance, as they are responsible for finger motion, the muscles of the upper arm can help improve the classification performance. Our results show a decrease in accuracy for our amputee subjects when we remove upper arm EMG data by 10% on average, see Fig. 5e. This outcome is aligned with the findings of [20], where it is shown that the activation of the proximal muscles is statistically different when the arm reaches to grasp objects with different characteristics or orientations. Although our experimental protocol constrains subjects to a single-hand orientation, the decreased accuracy when removing the upper-arm EMG indicates that the proximal muscles are important for classification accuracy during reaching.

In addition to the importance of the proximal muscles, we notice an improvement on the performance of the individuals who undergo a TMR operation. More specifically, the classification accuracy on the TMR subjects becomes better than the non-TMR subject on the second motion phase, whereas it stays at the same level of performance in the third phase. These results are aligned with studies [8, 9] that indicated the potential benefits of TMR on the classification accuracy. However, considering the small sample size of the group in our study, these results should be taken with a grain of salt. The improvements that TMR operations could provide on the performance of a myoelectric pattern-recognition system should be further investigated.

We compare four different classification methods, but find no significant differences in classification performance. LDA perform well: delivering similar results with SVM with either a linear or RBF kernel. The performance of the Echo State Network was on a level similar to the other classification methods. It is worth mentioning that no feature extraction is performed on the EMG before being inserted in the ESN. In this case, we let the random reservoir select the features and then train a linear regressor for classification. This indicates that a random projection of the EMG signals to a very high dimensional space could be sufficient for achieving good classification results.

This work presents an approach to improve the reaction time of a hand prosthetic device through a systematic assessment of the accuracy of a myoelectric pattern recognition system over different phase periods during reach-to-grasp motions. The EMG signals are collected from seven muscles of the upper arm and five muscles of the forearm, as we focus on a potential application for individuals with transradial amputation. Our approach could potentially be implemented for proximal amputations in cases where the user has an enhanced ability to control a myoprosthesis, e.g. after undergoing a TMR operation. TMR has been shown to increase the accuracy of a myoelectric pattern-recognition system also for the case of transhumeral amputation [9, 44]. This improvement increases the number of degrees of freedom that individuals with transhumeral amputation can control. A potential extension of our approach to a proximal upper-limb amputation could be possible for individuals with TMR. This extension would however require further work to model the activation of the residual muscles of the upper arm during reach-to-grasp motions and to select a smaller number of grasp types, for increasing the classification confidence.

An important extension of our approach is the introduction of a robotic control scheme that derives from the natural motion of the human hand, and that imparts a human-like behavior to the prosthesis. As the EMG activation is significantly different among the three phases of the motion, a combination of different classifier for each phase, or a combination of those, could improve the classification performance. This approach could also be applied in conjunction with a synergistic closure of the hand [28], tackling the problem of the high dimensionality of the task. A direct extension of this work could be the coupling of the closure of the hand with the motion of the arm; this could provide a more natural coordination between the hand and the arm. The introduction of different hand orientations and an additional wrist control could be a further expansion of the approach.

Another interesting extension of the proposed approach could be the introduction of the kinematics of the arm, towards a multi-sensor pattern-recognition system. In this case, the angular position or velocity could correspond to a parameter of the system, providing information regarding the phase of the motion and potentially improving the accuracy of the system.

## Conclusion

In this work, we have presented an electromyography-based approach for decoding the grasping intention during reach-to-grasp motions. Four able-bodied subjects and four individuals with transradial amputation participated in our study. In order to examine the evolution of the classification accuracy over the reach-to-grasp motion, we separated the motion into three phases: (1) the first phase- where the velocity of the motion increases, (2) the second phase- where the velocity of the motion decreases and (3) the third phase- when the reaching motion is complete. Our results have shown that it is possible to decode the grasping intention before the end of the reaching motion, especially during the second motion phase. The inclusion of the muscular activity of the upper arm to the pattern recognition algorithm increases its accuracy by 10% on average. As a proof of concept, we have evaluated our approach with an individual with a transradial amputation controlling a myo-prosthesis in real-time. The real-time evaluation shows a significant improvement in classification accuracy as well as in the reaction time of the device when all the motion phases are included in the training data. Further extensions should involve the evaluation of our proposed approach in in real-life conditions as well as with different hand’s orientations.

## Additional files

Additional file 1 The supplementary materials contain the activity of the selected muscles as well as results regarding the models of the motion phases for all the subjects. (DOCX 4358 kb)

## References

[1] Freeland AE, Psonak R. Traumatic below-elbow amputations. Orthopedics. 2007. 10.3928/01477447-20070201-16 17323634

[2] Raichle KA, Hanley MA, Molton I, Kadel NJ, Campbell K, Phelps E, Ehde D, Smith DG. Prosthesis use in persons with lower- and upper-limb amputation. J Rehabil Res Dev. 2008. 10.1682/jrrd.2007.09.0151 PMC2743731 19165686

[3] Need-Directed Design of Prostheses and Enabling Resources in Amputation, Prosthesis Use, and Phantom Limb Pain: An Interdisciplinary Perspective. New York: Springer; 2010.

[4] Oltstlie k, Lesjo IM, Franklin RJ, Garfelt B, Skjeldal OH, Magnus P. Prosthesis rejection in acquired major upper-limb amputees: a population-based survey. Disabil Rehabil. 2012. 10.3109/17483107.2011.635405 22112174

[5] Cordella F, Ciancio AL, Sacchetti R, Davalli A, Cutti AG, Guglielmelli E, Zollo L. Literature review on needs of upper limb prosthesis users. Front Neurosci. 2016. 10.3389/fnins.2016.00209 PMC4864250 27242413

[6] Novak D, Riener R. A survey of sensor fusion methods in wearable robotics. Robot Auton Syst. 2014.

[7] Earley EJ, Hargrove LJ, Kuiken TA. Dual window pattern recognition classifier for improved partial-hand prosthesis control. Front Neurosci. 2016. 10.3389/fnins.2016.00058 PMC4763088 26941599

[8] Miller LA, Stubblefield KA, Lipschutz RD, Lock BA, Kuiken TA. Improved myoelectric prosthesis control using targeted reinnervation surgery: A case series. IEEE Trans Neural Syst Rehabil Eng. 2008. 10.1109/TNSRE.2007.911817 PMC4318638 18303805

[9] Kuiken TA, Li BA, Guanglin amd Lock, Lipschutz RD, Miller LA, A SK, Englehart K. Targeted muscle reinnervation for real-time myoelectric control of multifunction artificial arms. JAMA. 2009. 10.1001/jama.2009.116 PMC3036162 19211469

[10] Urbanchek MG, Wei B, Baghmanli Z, Sugg K, Cederna PS. Long-term stability of regenerative peripheral nerve interfaces. Plast Reocnstractive Surg. 2011.

[11] Fougner AL, Stavdahl O, Kyberd PJ. System training and assessment in simultaneous proportional myoelectric prosthesis control. J Neuroengineering Rehabil. 2014. 10.1186/1743-0003-11-75 PMC4041142 24775602

[12] Young AJ, Smith LH, Rouse EJ, Hargrove LJ. Classification of simultaneous movements using surface emg pattern recognition. IEEE Trans Biomed Eng. 2013. 10.1109/TBME.2012.2232293 PMC4208826 23247839

[13] Smith LH, Kuiken TA, Hargrove LJ. Evaluation of linear regression simultaneous myoelectric control using intramuscular emg. IEEE Trans Biomed Eng. 2016. 10.1109/TBME.2015.2469741 PMC4761337 26302506

[14] Gonzalez-Vargas J, Dosen S, Amsuess S, Yu W, Farina D. Human-machine interface for the control of multi-function systems based on electrocutaneous menu: Application to multi-grasp prosthetic hands. PLoS ONE. 2015. 10.1371/journal.pone.0127528 PMC4466571 26069961

[15] Li G, Schultz AE, Kuiken TA. Quantifying pattern recognition-based myoelectric control of multifunctional transradial prostheses. IEEE Trans Neural Syst Rehabil Eng. 2010. 10.1109/TNSRE.2009.2039619 PMC3024915 20071269

[16] Naik GR, Al-Timemy AH, Nguyen HT. Transradial amputee gesture classification using an optimal number of semg sensors: An approach using ica clustering. IEEE Trans Neural Syst Rehabil Eng. 2016. 10.1109/TNSRE.2015.2478138 26394431

[17] Khushaba RN, Kodagoda S, Takruri M, Dissanayake G. Toward improved control of prosthetic fingers using surface electromyogram (emg) signals. Expert Syst Appl. 2012.

[18] Scheme E, Biron K, Englehart K. Improving myoelectric pattern recognition positional robustness using advanced training protocols. In: 33rd Annual International Conference of the IEEE EMBS. Boston: 2011. 10.1109/IEMBS.2011.6091196 22255419

[19] Batzianoulis I, El-Khoury S, Pirondini E, Coscia M, Micera S, Billard A. Emg-based decoding of grasp gestures in reaching-to-grasping motions. Robot Auton Syst. 2017.

[20] Martelloni C, Carpaneto J, Micera S. Characterization of emg patterns from proximal arm muscles during object- and orientation-specific grasps. IEEE Trans Biomed Eng. 2009. 10.1109/TBME.2009.2026470 19605312

[21] Rand MK, Shimansky YP, Stelmach ABMI, Stelmach HE. Quantitative model of transport- aperture coordination during reach-to-grasp movements. Exp Brain Res. 2008. 10.1007/s00221-008-1361-5 18438652

[22] Wang J, Stelmach G. E. Coordination among the body segments during reach-to-grasp action involving the trunk. Exp Brain Res. 1998. 10.1007/s002210050578 9860274

[23] Jeannerod M. The timing of natural prehension movements. J Motor Behav. 1984. 10.1080/00222895.1984.10735319 15151851

[24] Haggard P, Wing A. Coordinated responses following mechanical perturbation of the arm during prehension. Exp Brain Res. 1995. 10.1007/BF00230652 7737394

[25] Ghazaei G, Alameer A, Degenaar P, Morgan G, Nazarpour K. Deep learning-based artificial vision for grasp classification in myoelectric hands. J Neural Eng. 2017. 10.1088/1741-2552/aa6802 28467317

[26] Amsuess S, Vujaklija I, Goebel P, Roche AD, Graimann B, Aszmann OC, Dario F. Context-dependent upper limb prosthesis control for natural and robust use. IEEE Trans Neural Syst Rehabil Eng. 2016. 10.1109/TNSRE.2015.2454240 26173217

[27] Farrell TR, Weir RF. The optimal controller delay for myoelectric prostheses. IEEE Trans Neural Syst Rehabil Eng. 2007. 10.1109/TNSRE.2007.891391 PMC8173529 17436883

[28] Santello M, Soechting JF. Gradual molding of the hand to object contours. J Neurophys. 1998. 10.1152/jn.1998.79.3.1307 9497412

[29] Smith LH, L J Hargrove TAK B A Lock. Determining the optimal window length for pattern recognition-based myoelectric control: Balancing the competing effects of classification error and controller delay. IEEE Trans Neural Syst Rehabil Eng. 2011. 10.1109/TNSRE.2010.2100828 PMC4241762 21193383

[30] Englehart K, Hudgins B. A robust, real-time control scheme for multifunction myoelectric control. IEEE Trans Biomed Eng. 2003. 10.1109/TBME.2003.813539 12848352

[31] Daley H, Englehart K, Hargrove L, Kuruganti U. High density electromyography data of normally limbed and transradial amputee subjects for multifunction prosthetic control. J Electromyogr Kinesiol. 2012. 10.1016/j.jelekin.2011.12.012 22269773

[32] Jaeger H. The “echo state” approach to analyzing and training recurrent neural networks. Technical report, German National Research Institute for Computer Science. 2001.

[33] Li D, Han M, Wang J. Chaotic time series prediction based on a novel robust echo state network. IEEE Trans Neural Netw Learn Syst. 2012. 10.1109/TNNLS.2012.2188414 24806127

[34] Xu M, Han M. Adaptive elastic echo state network for multivariate time series prediction. IEEE Trans Cybern; 46(10):2173–2183. 10.1109/TCYB.2015.2467167 27455531

[35] Lenzi T, Lipsey J, Sensinger JW. The ric arm- a small anthropomorphic transhumeral prosthesis. IEEE Trans Mechatron. 2016.

[36] He J, Zhang D, Jiang N, Sheng X, Farina D, Zhu X. User adaptation in long-term, open-loop myoelectric training: implications for emg pattern recognition in prosthesis control. J Neural Eng. 2015. 10.1088/1741-2560/12/4/046005 26028132

[37] Peerdeman B, Boere D, Witteveen H, Veld RH, Hermens H, Stramigioli S, Rietman H, Veltink P, Misra S. Myoelectric forearm prostheses: State of the art from a user-centered perspective. J Rehabil Res Dev. 2011. 10.1682/jrrd.2010.08.0161 21938658

[38] Y Geng YW O W Samuel, Li G. Improving the robustness of real-time myoelectric pattern recognition against arm position changes in transradial amputees. BioMed Res Int. 2017. 10.1155/2017/5090454 PMC5421097 28523276

[39] J Liu XS, D Zhang, Zhu X. Quantification and solutions of arm movements effect on semg pattern recognition. Biomed Signal Process Control. 2014.

[40] Yang D, Jiang L, Osborn L, Gu Y, Liu H. Dynamic training protocol improves the robustness of pr-based myoelectric control. Biomed Signal Process Control. 2017.

[41] Wing AM, Turton A. Grasp size and accuracy of approach in reaching. J Mot Behav. 1986. 10.1080/00222895.1986.10735380 15138146

[42] Jeannerod M, Paulignan PWY. Grasping an object: one movement, several components. In: Novartis Foundation Symposium. New Jersey: Wiley. 10.1002/9780470515563.ch2 9949813

[43] T Supuk TBTKodek. Estimation of hand preshaping during human grasping. Med Eng Phys. 2005. 10.1016/j.medengphy.2005.03.008 16171739

[44] Hargrove LJ, Turner K, Kuiken TA, Miller LA. Myoelectric pattern recognition outperforms direct control for transhumeral amputees with targeted muscle reinnervation: A randomized clinical trial. Sci Rep. 2017. 10.1038/s41598-017-14386-w PMC5653840 29062019

[45] Gentilucci M, Benuzzi F, Bertolani L, Daprati E, Gangitano M. Language and motor control. Exp Brain Res. 2000. 10.1007/s002210000431 10985682

[46] Bongersa RM, Zaala FT, Jeannerod M. Hand aperture patterns in prehension. Hum Mov Sci. 2012. 10.1016/j.humov.2011.07.014 22130470

[47] Feix T, J Romero H-BS, Dollar AM, Kragic D. The grasp taxonomy of human grasp types. IEEE Trans Hum Mach Syst. 2015.

