# Supplementary material for: Decoding the grasping intention from electromyography during reaching motions
Source: J Neuroeng Rehabil. 2018 Jun 26;15:57. doi: 10.1186/s12984-018-0396-5 (PMC6020187; doi:10.1186/s12984-018-0396-5)
Supplement: Supplementary file 2 — The supplementary materials contain the activity of the selected muscles as well as results regarding the models of the motion phases for all the subjects. (DOCX 4358 kb) [file 12984_2018_396_MOESM1_ESM.docx]

**Supplementary materials for**

Decoding the grasping intention from Electromyography during reaching motions

Iason Batzianoulis, Nili E. Krausz, Ann M. Simon, Levi Hargrove and Aude Billard

correspondence to: [iason.batzianoulis@epfl.ch](mailto:iason.batzianoulis@epfl.ch)

**This file includes:**

Supplementary Results

**Other Supplementary Materials for this manuscript includes the following:**

The video “demo_jner.mp4”

**Sepplementary Methods**

In this section, we present the location of the electrodes for each recorded muscle (see Figure s1).
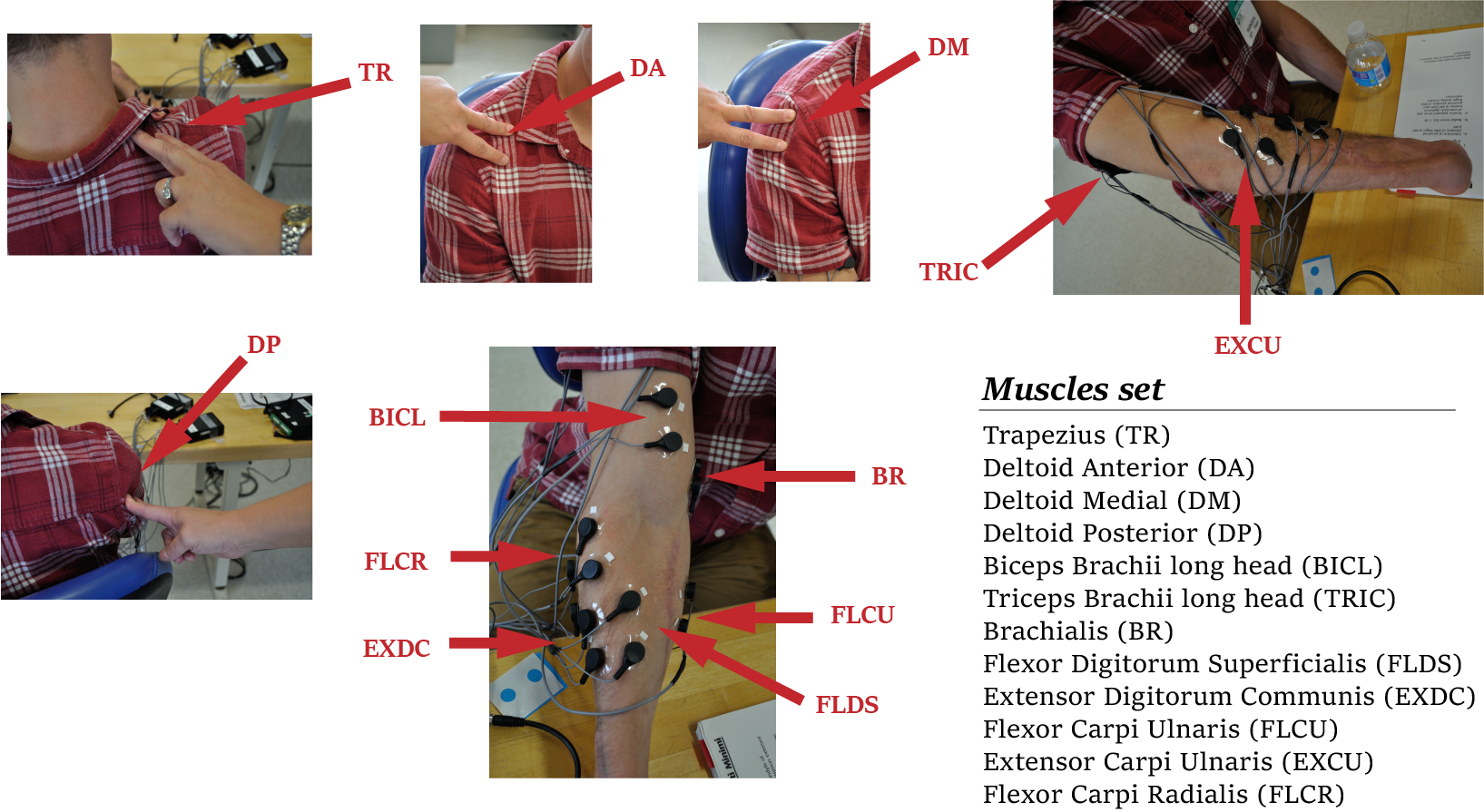


Figure s1: The location of the electrodes and the muscles set of this study

**Sepplementary Results**

In this section, we present additional results regarding the muscular activity of the motion phases.


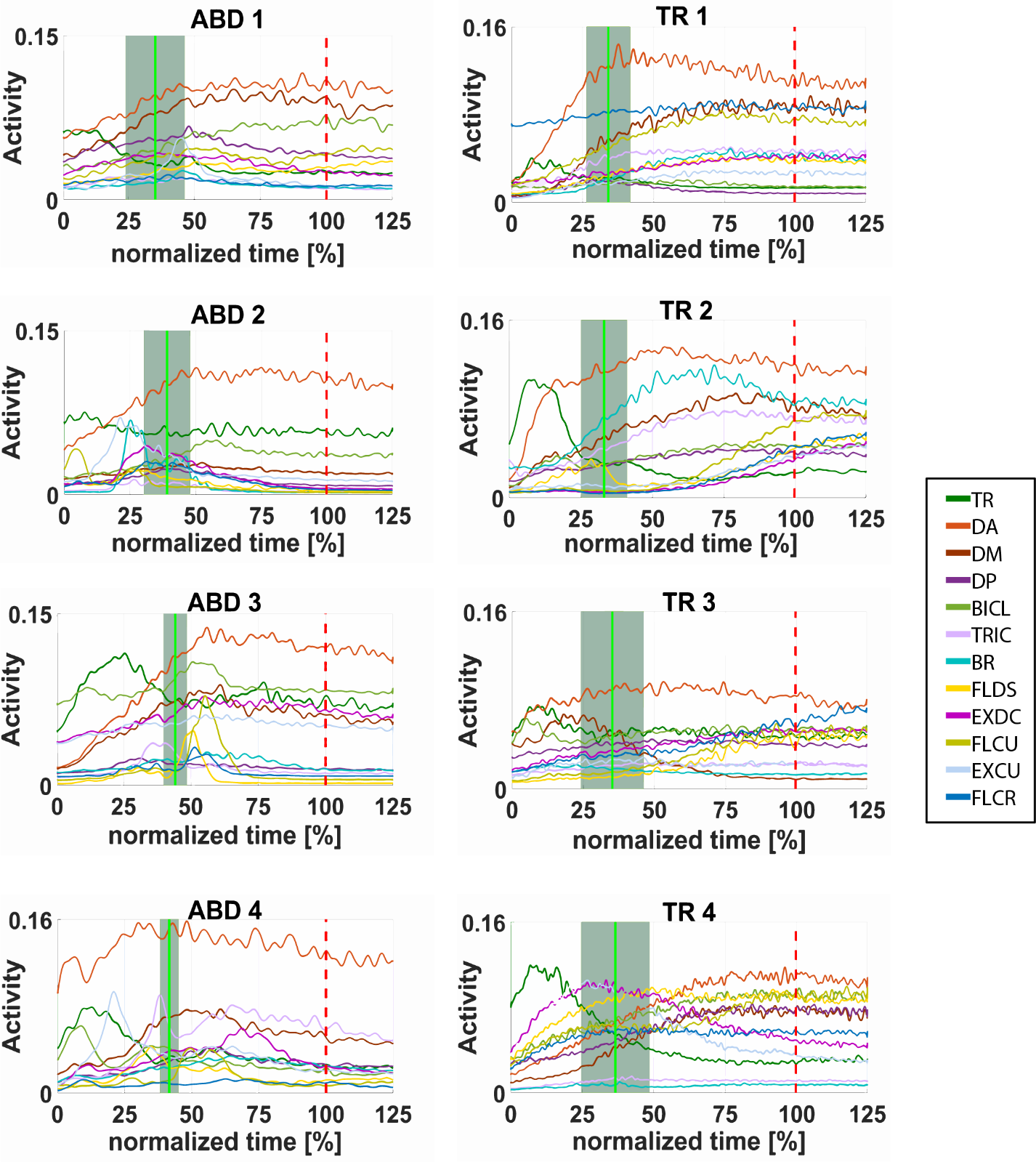


Figure s2: The linear envelope of the EMG signals of the able-bodied subjects (left) and amputee subjects (right).

*Figure s2* presents the average EMG activity the muscles of each group in normalized time. The green vertical like shows the moment that the angular velocity of elbow joint reached its maximum, while the shaded area around it corresponds to its standard deviation of the moment the maximum velocity occurred. The activation of the more distal muscles in able-bodied subjects occurs in an earlier phase than in amputees. The muscular activity of the forearm muscles of ABD 2, 3 and 4 is reaching its peak from 20-60% of the motion, while only the forearm muscles of ABD5 and TR4 are activating in the early stages of the reaching motion. Moreover, the EMG activity of the forearm muscles is decreasing as the motion is getting closer to completion on the able-bodied subjects. On the other hand, the EMG activity of forearm muscles of the amputee subjects increases, either gradually during the reaching motion (TR1,TR3, TR4-main text) or more rapidly in the end of the motion (TR2). The different timing of activation could have an impact to the classification performance as we see later in the document. The more proximal muscles stay in the same level of activation after the maximum angular velocity is reached.


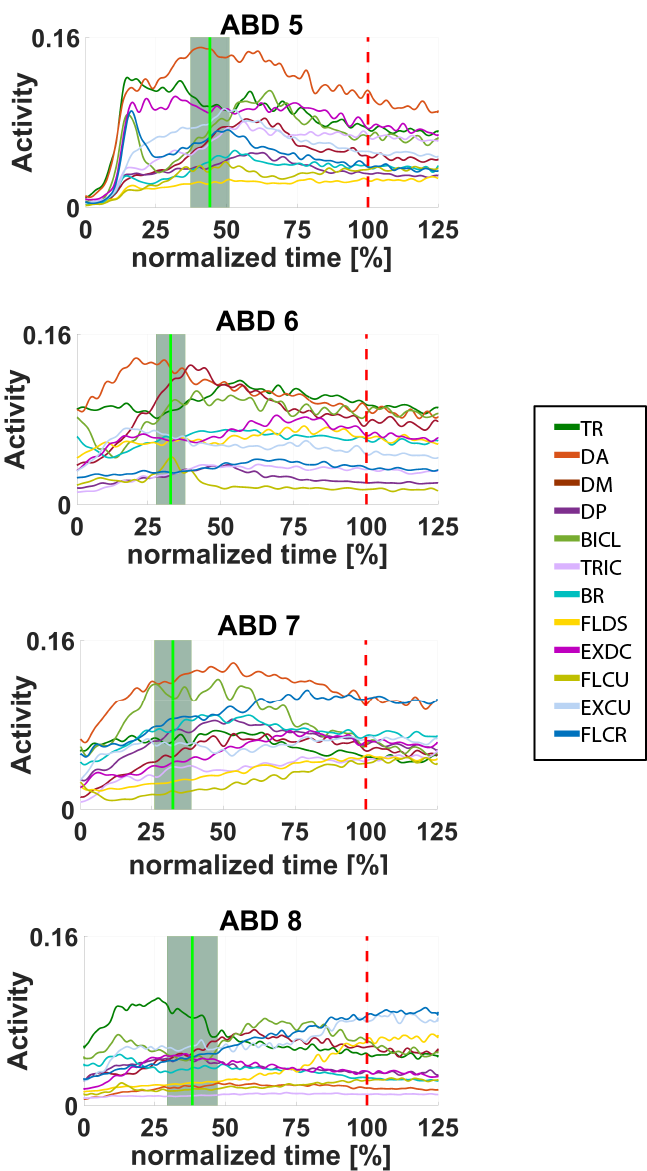


Figure s3: The linear envelope of the EMG signals of able-bodied subjects 5-8.


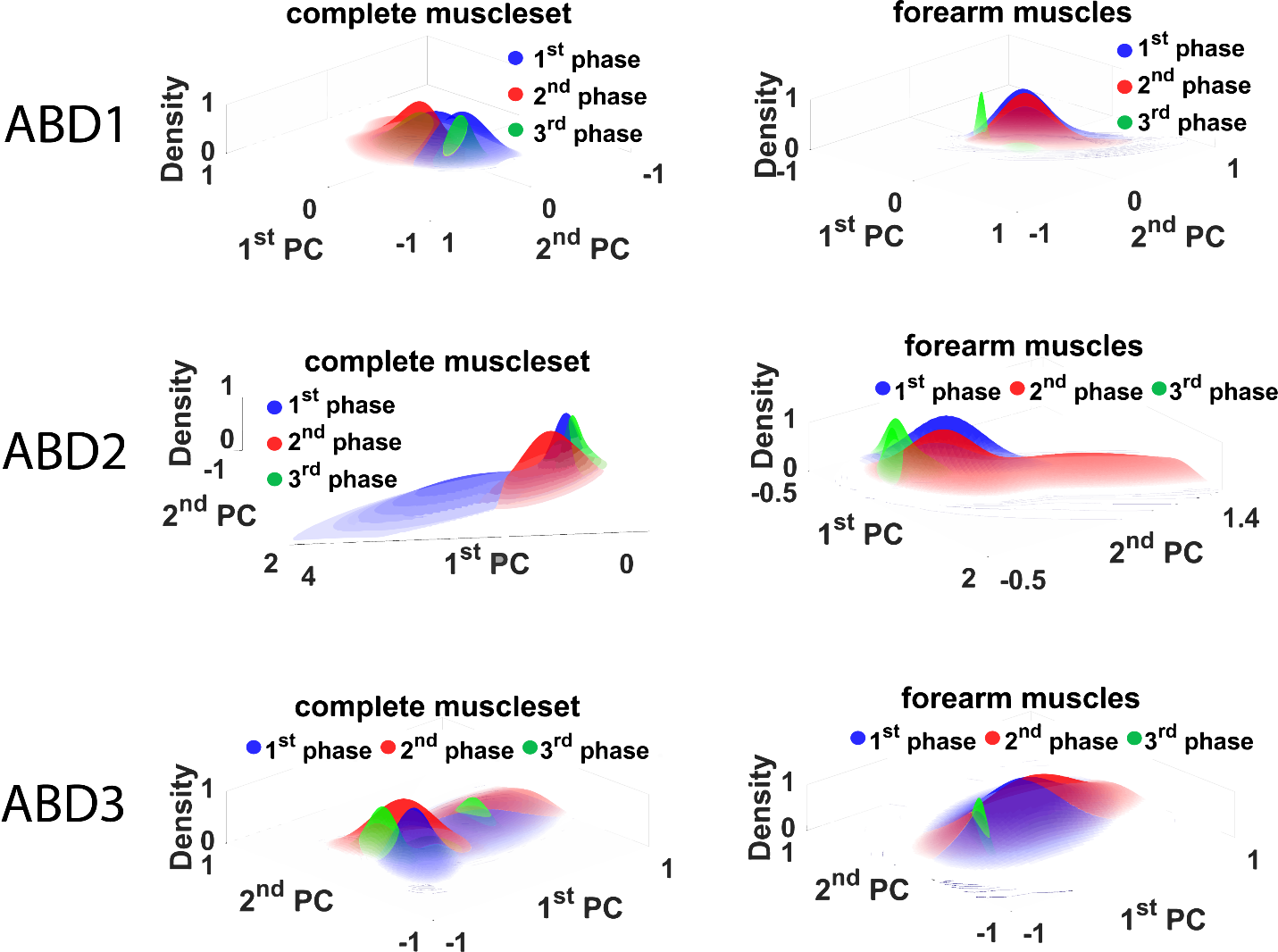


Figure s4: A representation with Gaussian Mixture Models (GMMs) of the EMG activity of the three phases projected on the first two components of third phase after performed Principal Component Analysis (PCA). The analysis was performed on the complete muscle set (N = 12) and when using only the muscles of the forearm (N = 5). The blue color corresponds to the first phase, the red color to the second phase and the green color to the third phase. The results presented in this figure regard the EMG activity of able-bodied subjects 1-3.

*Figures s3*, *s4* and *s5* present the Gaussian models of the phases on the first two principal components for the complete muscle-set and the muscles of the forearm respectively for able-bodied (*Figures s3* and *s4*) and amputee subjects (*Figure s5*). Although some models are partially overlapping, the means of the models are different with each other in all the subjects, regardless the muscle-set. In able-bodied subjects (*Figure s3* and *s4*), the models of the third phase are concentrated on the area around the origin and their corresponding standard deviations are smaller than the standard deviation of the models of the other two phases. The larger overlapping was noticed on the models of first and third phases for the complete muscle-set of the subjects ABD1 and ABD2 and between all the models of the forearm muscles of ABD3. While the models of the third phase are concentrated around the origin, similarly to the able-bodied subjects, they cover a larger area than the corresponding models of the able-bodied subjects. A larger overlapping area is noticed between the models of the first and second phase for all the amputee subjects, while the models of the third phase are more distant form the other two.


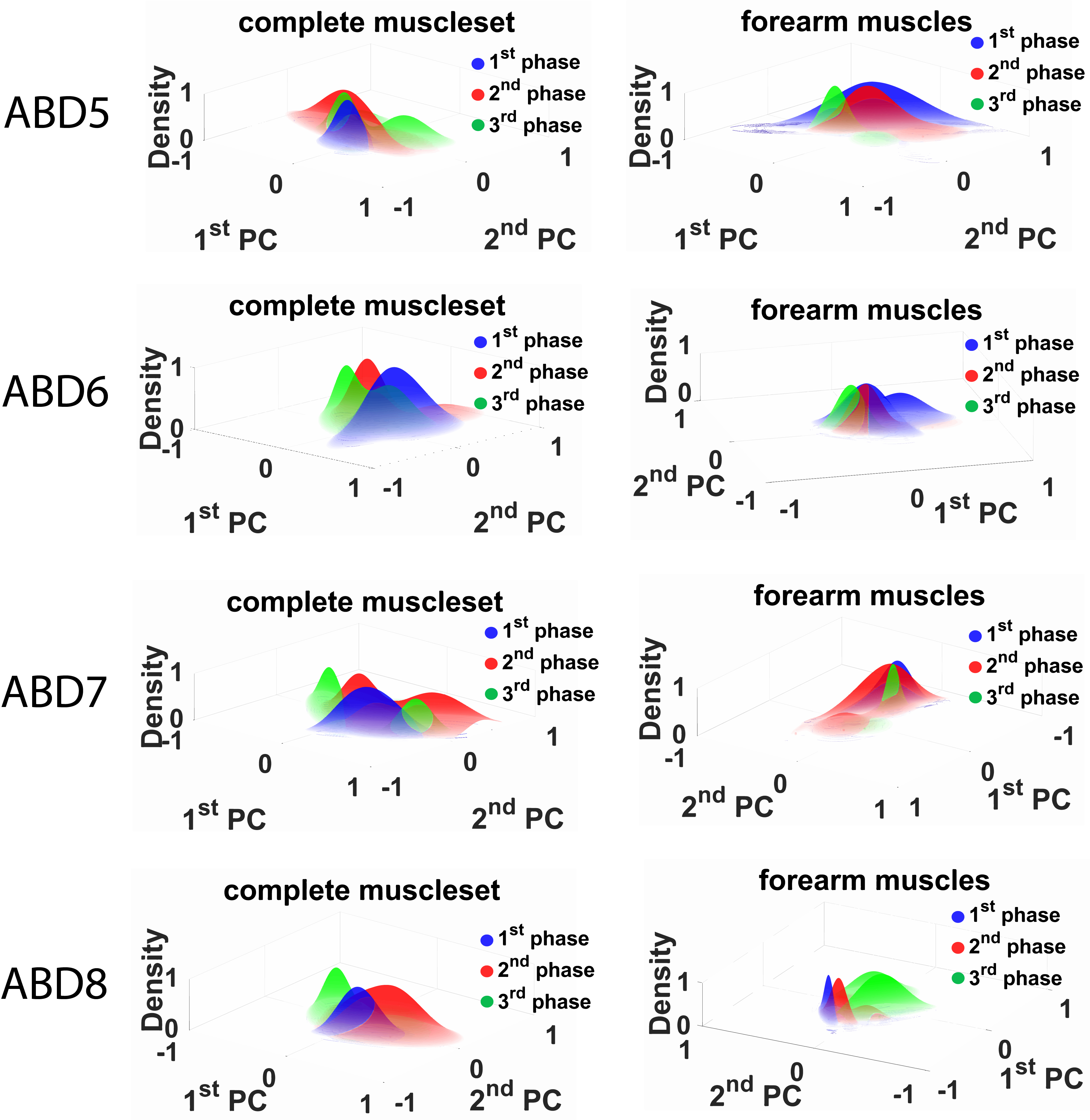


Figure s5 A representation with Gaussian Mixture Models (GMMs) of the EMG activity of the three phases projected on the first two components of third phase after performed Principal Component Analysis (PCA). The analysis was performed on the complete muscle set (N = 12) and when using only the muscles of the forearm (N = 5). The blue color corresponds to the first phase, the red color to the second phase and the green color to the third phase. The results presented in this figure regard the EMG activity of able-bodied subjects 1-3.


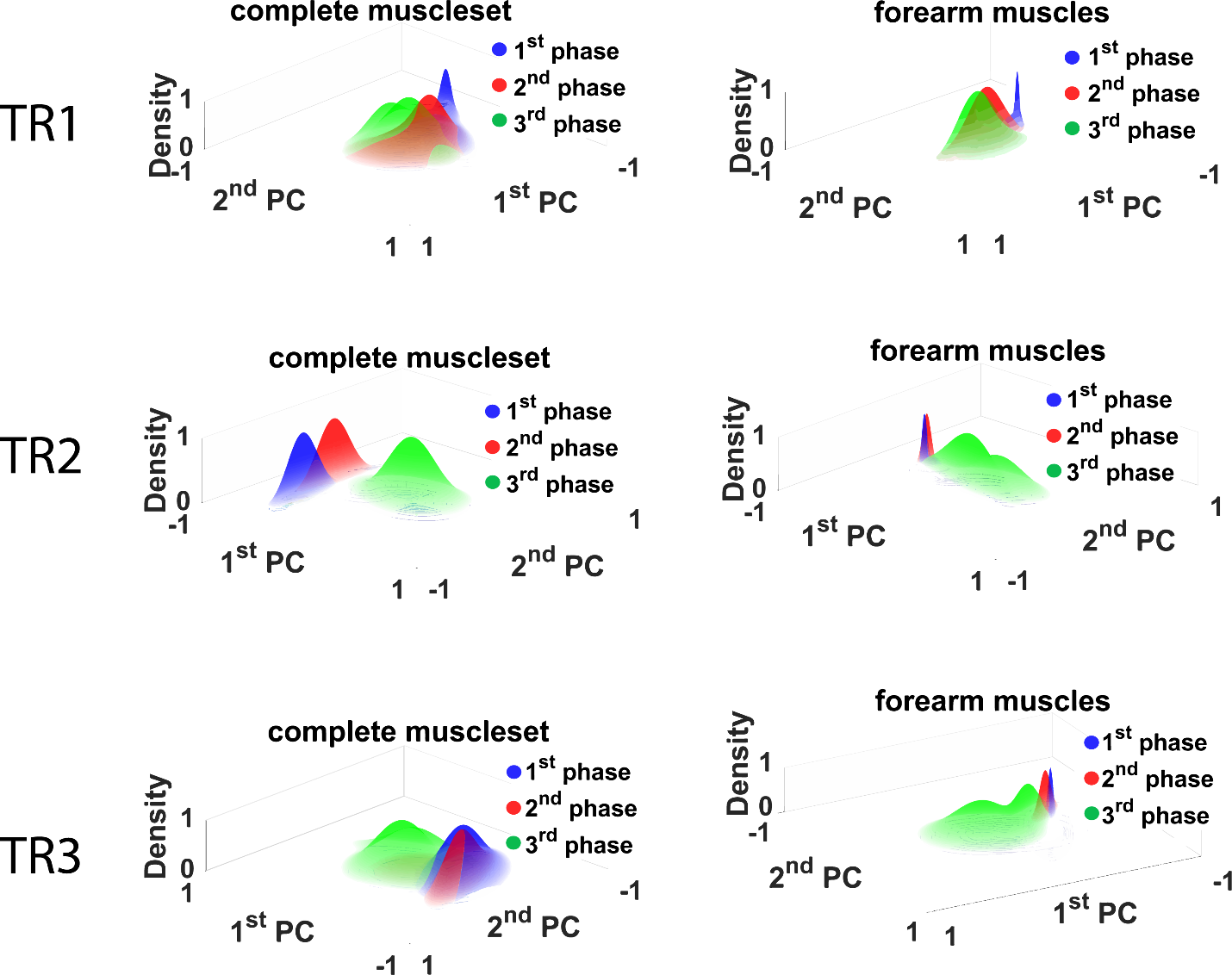


Figure s6 A representation with Gaussian Mixture Models (GMMs) of the EMG activity of the three phases projected on the first two components of third phase after performed Principal Component Analysis (PCA). The analysis was performed on the complete muscle set (N = 12) and when using only the muscles of the forearm (N = 5). The blue color corresponds to the first phase, the red color to the second phase and the green color to the third phase. The results presented in this figure regard the EMG activity of amputee subjects.

Similar results are noticed regarding the phases of the motion for the amputee subjects (*Figure s4*). More particularly, the models of the second and third phase are partially overlapping for TR2 and TR3. The models of the third phase of the forearm muscles of TR2 and TR3 occupy a larger area than the other two, while the models occupy approximately equal space when the complete muscle-set in taken into account. Regarding the subject TR1, the models of the second and third phase are overlapping more extensively than the other subjects.

**Video demo_jner.mp4**

The video presents the motivation of this study, the experimental protocol of the offline analysis as well as an illustration of the models of motion phases and the real time evaluation of the approach.
